# The Risks of Renal Angiomyolipoma: Reviewing the Evidence

**DOI:** 10.15586/jkcvhl.2017.97

**Published:** 2017-10-16

**Authors:** Raouf M. Seyam, Waleed K. Alkhudair, Said A. Kattan, Mohamed F. Alotaibi, Hassan M. Alzahrani, Waleed M. Altaweel

**Affiliations:** Department of Urology, King Faisal Specialist Hospital and Research Center, Riyadh, Saudi Arabia

**Keywords:** angiomyolipoma, embolization, hemorrhage, kidney, nephrectomy

## Abstract

Renal angiomyolipoma (RAML), though a rare benign tumor, may impose a significant morbidity or even mortality due to its unique characteristics and the complications subsequent to its treatment. The classic tumor variant is composed of smooth muscular, vascular, and fatty components. The most straightforward diagnosis is when the fat component is abundant and gives a characteristic appearance on different imaging studies. In fat-poor lesions, however, the diagnosis is difficult and presumed a renal cell carcinoma. Yet, some variants of RAML, though rare, express an aggressive behavior leading to metastasis and mortality. The challenge lies in the early detection of benign variants and identifying aggressive lesions for proper management. Another challenge is when the vascular tissue component predominates and poses a risk of hemorrhage that may extend to the retroperitoneum in a massive life-threatening condition. The predicament here is to identify the characteristics of tumors at risk of bleeding and provide a prophylactic treatment. According to the clinical presentation, different treatment modalities, prophylactic or therapeutic, are available that span the spectrum of observation, embolization, or surgery. Renal impairment may result from extensive tumor burden or as a complication of the management itself. Improvement of diagnostic techniques, super-selective embolization, nephron-sparing surgery, and late treatment with the mammalian target of rapamycin inhibitors have provided more effective and safe management strategies. In this review, we examine the evidence pertaining to the risks imposed by RAML to the patients and identify merits and hazards associated with different treatment modalities.

## Introduction

Renal angiomyolipoma (RAML) is a rare benign tumor that has acquired much attention because of the risks it poses to patients and sometimes due to its ability to mimic renal cell cancer (RCC) (1–5). Sporadic RAMLs (sRAML) are smaller than their tuberous sclerosis (TSC)-associated counterparts, usually unilateral and risk bleeding only when large. The indications of treatment in symptomatic patients include hemorrhage and pain. Asymptomatic patients are treated to mitigate the risk of bleeding, for the suspicion of malignancy, and to prevent renal impairment. The management of RAML has changed over the years: while early treatment was nephrectomy, later a more conservative approach utilizing embolization and nephron sparing surgery (NSS), and finally the mammalian target of rapamycin (mTOR) inhibitors has been proved effective to halt and reverse TSC-associated RAML (TSC-RAML) (2, 3). The caveats of intervention are mainly loss of renal tissue and function, while mTOR inhibition is effective only during the time of treatment. RAML itself or its treatment may cause significant morbidity and mortality to patients. In this article, we examine the evidence supporting the concerns raised by the medical community that justify prophylactic treatment or intervention. Meanwhile, we review the efficacy and safety of management, whether prophylactic or therapeutic.

## Risk of Bleeding

Emergency treatment of bleeding associated with RAML continues to be a challenge to save the patient while preserving renal function. Several studies concluded that large lesions >4 cm and TSC-associated lesions are more susceptible to bleeding, raising the need for prophylactic management.

### Acute bleeding

The most common cause of retroperitoneal bleeding of non-traumatic renal origin, sometimes responsible for fatality, is RAML followed by RCC (6, 7). Retroperitoneal hemorrhage is associated with large lesions (1, 8), spontaneous rupture of RAML (9), rupture of aneurysms (10, 11), associated with pregnancy (12, 13), trauma even trivial (14–16) and anticoagulation (17, 18). The management of bleeding RAML initially was nephrectomy (1, 5). A 10-year experience in the treatment of RAML favored nephrectomy or partial nephrectomy (PN) in 71.2% of patients and embolization in 28.8% (5). As technology evolved, embolization became the treatment of choice, leaving nephrectomy only for failed embolization in the emergency setting, development of post-embolization complications, and life-threatening hemorrhage (19–21). Embolization is associated with a high clinical success rate more than 90% (22, 23). Successful control of a bleeding emergency was reported in 96–100% of patients (20, 24). Bleeding occurs in both sporadic and TSC-associated lesions. A pooled analysis identified 441 patients with sRAML with a mean initial lesion diameter of 6.5 cm (25). Major retroperitoneal bleeding occurred in 12.2% of the patients, and the intervention rate was high for the whole group including 58.1% nephrectomy or PN, 29% embolization, and 7.3% conservative treatment. Active treatment was frequently reported (67.5%) in 400 patients with RAML, whereas the remaining 130 patients, 78.5% of whom were asymptomatic, underwent active surveillance (26). There is a consensus that symptomatic RAML mainly due to hemorrhage should be treated by embolization and surgical excision as a second-line treatment (27, 28).

### Prophylaxis against bleeding

There is controversy regarding prophylactic management of asymptomatic RAML. Active surveillance in patients with small lesions (mean size 1.7 cm) was associated with low growth rate (0.088 cm/year) and minimal (7%) secondary intervention (29). Larger lesions may be at high risk of bleeding, but there is no consensus on what size should be used as a cutoff for prophylaxis (27). Although in the pediatric age group, the incidence of RAML in TSC patients is 50.3%, growth rates are unpredictable and require only radiological follow-up (30). In that study, while management of the renal lesions in 145 patients was not consistent, fortunately none of the patients developed retroperitoneal hemorrhage.

Several options are available for prophylaxis, ranging from observation up to surgery (Table 1). The most drastic prophylaxis is total nephrectomy for large lesions (5) (Supplementary Table 2; the supplementary tables of this article are available on the journal’s website, which can be accessed via the following link: http://jkcvhl.com/index.php/jkcvhl/rt/suppFiles/97/0). A surgeon’s preference might be driven by the less demanding procedure compared to PN. Guidelines recommend that nephrectomy in TSC patients should be avoided (31). Prophylactic PN is contemplated by urologists as it provides a “permanent cure” of the resectable lesions, can be performed using minimally invasive surgery either laparoscopic or robotic assisted, and is associated with preservation of renal function (4, 5, 29, 32–41) (Supplementary Table 2). Surgical complications up to 21% were reported (5, 32, 33, 35, 36, 38). Only one patient out of 132 in four studies developed renal failure in a follow-up of 26 months to 8 years (32, 33, 36). No recurrence or a maximum recurrence rate of 3.4% developed at long-term follow-up (29, 32, 33, 35, 36, 38). The most recent advent of NSS is robotic PN (RPN). Three series included prophylactic RPN reported mostly low-grade complications, preservation of renal function, and no recurrence of lesions (39–41). Prophylactic embolization of RAML has been widely used with 85–91% success rate (22, 42–45) (Supplementary Table 3). The aim of prophylactic treatment is to devascularize the lesion and reduce its size. Several studies reported a mean size reduction of 25–72% (46–49). No bleeding or need for surgical intervention was required in up to 17% of patients (8, 43, 44, 46, 49, 50). The caveat of this approach is the need of multiple retreatments (8, 22, 42, 43, 47, 50). The highest reported complication rate was 19.5%  (4, 8, 42, 44, 48). There is a risk of deterioration of the function of the treated renal unit; however, several studies showed no significant change in serum creatinine during a follow-up of 23–60 months (8, 44, 49, 50). Observation and active surveillance are viable options for patients who do not need active treatment (26, 29, 51–55) (Supplementary Table 4). Follow-up for 40–60 months was associated with no bleeding or size change (54, 55), growth in 8% (53), and need for intervention in 5.6–13% (26, 29, 52) or surgery in 29% (51). Observation for TSC-AML showed an association between severe hemorrhage and lesions >3.5 cm affecting 20% of patients prospectively followed for 60 months (56). Others showed a lower rate of hemorrhage and slow growth rate at 48 months (34). The studies of everolimus for the treatment of TSC-AML patients reported a significant reduction in lesion size and no bleeding at follow-up, rendering the medication an important prophylactic option (2, 57) (Supplementary Table 5).

**Table 1:** Summary of selected series which included prophylactic management against the risk of bleeding.

Method	Lesions	Follow-up duration (months)	Complications	Outcome	References
NSS	sRAMP/TSC-AML	15–96	0–21.4%	Preservation or renal function, rare renal impairment, recurrence 0–3.4%	(4, 5, 29, 32–38)
RPN	sRAMP/TSC-AML	8–40	15–26% including Clavien Grade I–II	eGFR preservation and no recurrence	(39–41)
Embolization	sRAMP/TSC-AML	20–85	multiple retreatments reported between 12 and 50%, complication rate varied between 0 and 19.5%	85–91% success rate, a mean size reduction of 25–72%, no bleeding or need for surgical intervention was required in 0–17%, no significant change of serum creatinine	(4, 8, 22, 42–50)
Observation	sRAMP/TSC-AML	40–60		No bleeding or size change, growth in 8%, need for intervention in 5.6–13% or surgery in 29%	(26, 29, 51–55)
Everolimus	TSC-AML	9.5–29	2 patients discontinued treatment because of adverse events, adverse events mostly grades 1–2	42–54% clinical response (size reduction >50%), no bleeding reported in patients or controls	(2, 57)

AML, angiomyolipoma; eGFR, estimated glomerular filtration rate; NSS, nephron sparing surgery; RPN, robotic partial nephrectomy; sRAML, sporadic renal AML; TSC, tuberous sclerosis complex.

For asymptomatic patients, the European Association of Urology guidelines recommend surveillance and for high-risk patients embolization, PN, nephrectomy, or everolimus, albeit, with a weak evidence supporting any of these choices (27). High risk includes large lesion (size disputed), women in childbearing age and inadequate access to follow-up, or emergency service. For asymptomatic patients with TSC-RAML, guidelines from the 2012 TSC Consensus Conference recommended mTOR inhibitors as first-line short-term prophylactic treatment for patients with asymptomatic lesions >3 cm with category 1 recommendation (28). Second-line prophylactic procedures include embolization and NSS.

Several descending views challenge prophylaxis in asymptomatic patients. In patients with TSC, one study reported a slow growth rate of RAML, with a mean inclusion mass of 3.6 cm and a median follow-up of 4 years (34). Asymptomatic lesions <4 cm can be followed up conservatively and rarely will become complicated (53). Following patients, mostly asymptomatic, treated by active surveillance for a mean of 40 months, only 13% required active treatment (26). Risk factors for the need of delayed intervention were tumor size ≥4 cm and having symptoms at diagnosis. Even larger lesions in asymptomatic patients were successfully managed only by observation (54). Follow-up of 22 renal units in asymptomatic patients, with four lesions 4–10 cm in diameter, showed no bleeding and only one progression in size at a mean duration of 3.8 years.

Another study recommended only surveillance for all asymptomatic patients with sRAML regardless of size as the natural history of these lesions has not been well characterized and the annual growth rate of lesions is extremely slow (52). The analysis included patients with RAML (*n* = 447) who had three or more radiologic imaging. Regardless of size, RAML was stable with no appreciable growth at a median follow-up of 43 months. It is noteworthy, however, that the median size of the lesions was 1 cm and 90% of the patients had lesions ≤4 cm. In addition, of the 47 patients with lesions >4 cm, 38% (*n* = 18) had an intervention. From the larger cohort (*n* = 2741), seven patients with lesions >4 cm were excluded and had a nephrectomy or PN because of bleeding (*n* = 3), pain (*n* = 1), or electively (*n* = 3). Recalculating interventions for lesions >4 cm, 53.2% of patients required an intervention or surgery. From these data, one may conclude that more than 90% of patients with RAML ≤4 cm did not need an intervention, whereas more than half of patients with lesions >4 cm had an intervention or surgery.

### Pregnancy and risk of bleeding

RAML and pregnancy constitute a challenging situation. In the absence of a large series, the risk of bleeding remains not well defined. Case reports imply that an accelerated growth of RAML may occur during pregnancy and subsequent pregnancies may have a higher chance of hemorrhagic complications warranting prophylactic treatment (12, 13). Bleeding may prompt emergency treatment. Conservative management and elective caesarian section were reported when the patient was asymptomatic, hemodynamically stable, or could be stabilized by blood transfusions (58–61). Treating a bleeding lesion by nephrectomy or PN may compromise the continuation of pregnancy (62). Meanwhile, case reports of embolization and nephrectomy during pregnancy with uneventful outcome were reported (63, 64). A literature review found 21 cases of bleeding RAML managed during pregnancy in the past 35 years (62). The reported management included conservative treatment in eight women, embolization in five women, and nephrectomy in seven women. Related fetal death was reported in two cases. The hemorrhagic shock itself may lead to fetal demise (65). Few cases were reported where embolization was used to treat the pregnant mother (63, 64, 66). The risk of fetal exposure to radiation must be considered. Once the mother has reached full term, the preferred method of delivery is through caesarian section. Cases were reported of RAML rupture, retroperitoneal hemorrhage, and acute abdomen during or immediately after vaginal delivery (67, 68). One case was reported with uneventful course after vaginal delivery in a patient treated conservatively for RAML rupture (69). Other challenging special conditions were reported and successfully managed (70–74).

### mTOR inhibitors and bleeding

Several studies showed that mTOR inhibition results in a clinically significant reduction of TSC-RAML with acceptable tolerability and safety (2, 3, 75, 76). In 2012, everolimus was approved for the treatment of TSC-RAML. Treatment with everolimus for 1 year resulted in a reduction of the size of renal lesions by at least 50% in 53.3% of patients (77). These findings were confirmed in a clinical trial involving patients with TSC-RAML of at least 3 cm diameter (2). The clinical response rate was 42%. In an open label extension of the trial at a median follow-up of 29 months, the response rate increased to 54% (57). No patient developed bleeding from the kidney during the trial or its extension. A meta-analysis suggested that everolimus treatment prevented bleeding in those patients (78). As the risk of bleeding is associated with larger lesion, it could be inferred that treatment with everolimus will decrease the risk of bleeding by size reduction. However, there was no reported direct evidence that everolimus did decrease the risk of bleeding in TSC patients compared to controls (2). Being an oral medication that is generally well tolerated, everolimus is an attractive alternative for prophylaxis against renal hemorrhage with the caveats of adverse events (AE) related to its immunosuppression and metabolic effects (60). mTOR therapeutic effect is reversed after cessation of treatment (75). Recommendations regarding the long-term duration of treatment have not been established. Additional everolimus benefits that may tip the balance in its favor are its concomitant therapeutic effect on brain lesions and safety on renal function. Everolimus has not been approved for the treatment of sRAML regardless of its size or associated risks.

## Renal Malignancy

The issue of renal malignancy arises when a fat-poor AML is encountered, AML harboring RCC components is suspected of, or an epithelioid variant of the AML is present.

### Fat-poor AML

RAML has a characteristic radiologic appearance based on its fat content. A fat-poor renal mass is treated as RCC, usually by PN or total nephrectomy depending on surgical feasibility. The diagnosis of RAML in this situation is post surgery. In a large series of 730 patients, fat-poor AML constituted 4.8% of lesions <4 cm surgically excised with a preoperative diagnosis of renal cancer (79). In another large series of patients treated surgically, fat-poor AML was found in 33–65% of lesions, most commonly in small single lesions and least commonly in TSC-associated lesions (80). Consequently, preoperative diagnosis may prevent unnecessary surgery for a low-risk RAML. An attempt at preoperative diagnosis utilized a modification of imaging techniques and patient characteristics (79). Fat-poor AML tends to occur with smaller lesions, female gender, and younger age (79). Imaging modifications and novel image analysis try to demonstrate the presence of a minute amount of fat within the lesion. Such techniques include ultrasound (81), computed tomography (82, 83), and magnetic resonance imaging (MRI) (84–88). Preoperative diagnosis is possible by transcutaneous biopsy. The presence of fat, HMB-45 markers, or smooth muscle histologic markers discloses the diagnosis. The indications of special imaging or biopsy to rule out fat-poor RAML have not been well defined. When does the managing physician suspect AML rather an RCC for a solid renal lesion? We propose that such situations may include the association with TSC, the presence of multiple lesions, association with triphasic AML lesions, history of AML, and inconclusive radiological diagnosis.

### Renal cell carcinoma

The development of RCC within a RAML is rare but possible. Follow-up of mass growth, imaging characteristics, and biopsy may disclose the nature of the lesion and prompt proper management. Coexistent renal cancer and RAML was found in 34 cases (1%) in nephrectomy or PN specimens carried out for renal masses pooled from multiple centers (89). The association was found in both sporadic and TSC AML and the most common pathologic type was clear cell RCC. In a cross-sectional study of asymptomatic patients with TSC, 2.2% had RCC (90). Even in the pediatric age group, one patient out of 145 with TSC-RAML developed RCC (30). In another study, a total of 54 RCC lesions were reported in 18 patients with TSC (91). Most of the lesions (94%) were associated with AML. RCC was commonly multiple synchronous, metachronous, and/or bilateral. The histologic types were chromophobe RCC (59%), RCC with smooth muscle stroma (30%), and granular eosinophilic type. A distinctive feature of all RCC lesions is negative HMB-45 immunostaining. RCC was more common in females (13:^5^), developed at an early age, and had an indolent course at a mean follow-up of 52 months. An unusually high prevalence of concurrent RCC and RAML was reported in none-TSC patients. In a retrospective study of nephrectomy specimens for sRAML, concurrent RCC was found in 47% of lesions mostly of clear cell type (56%) (92).

### Epithelioid RAML

The diagnosis of epithelioid RAML is difficult because of the radiologic similarity to triphasic AML. Epithelioid AML (eAML) was reported in 7.7% of AML cases subjected to histopathologic diagnosis (93). eAML has an aggressive behavior, leading to metastasis, renal vein/inferior vena cava invasion, and in one study only 50% survival at 3 years (94). eAML has fewer or no fat content and is less frequently bilateral compared to classic AML. A study of a large series of patients with pure eAML reflected the aggressive behavior of the tumor in 41 patients (95). At the time of presentation, 79% patients were symptomatic and 12 (29.3%) patients had already metastasis. A follow-up of 33 patients (median 24.5 months) revealed recurrence in 17%, metastasis in 49%, and death in 33%. Another large series included 34 patients with eAML and atypia (96). Recurrence or metastasis was reported in 9 (26%) patients, four of whom died from the disease by 34 months. The rather aggressive behavior reported in these series might be related to histological characteristics of the lesions. The malignant behavior of eAML might be predicted by the proportion of epithelioid cells, tumor necrosis, the degree of nuclear atypia, and mitosis (93). Other studies showed a lower rate of aggressive behavior. In one study, only 5 (6.4%) patients with RAML had an epithelioid histopathology and only one developed metastasis (97). In another study, only one patient developed metastasis out of 11 patients with eAML (98). A lower rate of metastatic behavior was reported in a large series of 20 patients with renal eAML where only one (5%) developed metastasis (99). The malignant potential of eAML might be related to p53 mutation (97, 100). Preoperative diagnosis of eAML might be confused with RCC, particularly in cases which present with venous extension or metastasis (101). Several studies tried in retrospect to provide radiologic characteristics that may predict the diagnosis of eAML and differentiate it from classic triphasic AML and RCC (98, 102–104). Fine-needle aspiration (FNA) diagnosis of AML is difficult when the epithelioid type predominates. A group of lesions that were subjected to FNA subsequently proved AML on histopathology (105). The diagnosis was possible in only half the lesions, while in nearly half of the lesions FNA was nondiagnostic or erroneous. It remains to be seen that prospectively any radiologic characteristics or techniques can provide an accurate preoperative diagnosis of eAML.

For such a dismal tumor, it is noteworthy to mention that case reports of everolimus treatment of eAML showed a dramatic response of the lesion and metastasis (106, 107). The role of such a treatment needs to be defined.

## Renal Impairment

There is a plethora of evidence that associated renal impairment with RAML, particularly with TSC. In a cohort study, 16% of patients reached a chronic kidney disease (CKD) stage 3 or higher during a 12-year follow-up (108). A steeper yearly decline of glomerular filtration rate (GFR) was found in patients with TSC-RAML compared to the normal population (108). Age and RAML size correlated with CKD. The mortality rate was significantly higher in TSC patients (8.3%), mainly related to renal causes (2.6%), compared to the normal population (4.8%) (109). A meta-analysis confirmed that significantly more CKD is prevalent in TSC patients compared to age-matched population and there is a decrease in renal function as age increases (78). Moreover, renal causes accounted for 27.5% of the mortality reported in treated TSC patients (110). Several TSC patients have been treated in dialysis centers and some had high mortality (111, 112). Pediatric TSC patients may still develop renal insufficiency, though rarely (30).

Many review articles tried to explain the underlying pathology of renal impairment in patients with TSC (31, 113). Encroachment on the normal renal parenchyma or compression was provided as a possible mechanism. These conclusions were based on a few original articles (110–112).

In a review of causes of mortality in patients with TSC, 40 deaths related to TSC in 355 cases were reported (110). Renal causes accounted for 11 (27.5%) of these deaths. Two patients died from hemorrhage, two from renal malignancy, and seven from renal failure. The authors state that renal failure may have occurred because of angiomyolipoma, renal cysts, or both. Indeed, in an earlier report from the same group, 80% of the 15 patients who died had cystic lesions alone or with angiomyolipoma and three patients died from polycystic kidney disease (PCK) (114). In another study, of 65 TSC patients treated in dialysis centers in France, AML alone accounted for 23.1% of cases, renal cysts for 18.5% of cases, AML with renal cysts for 53.8% of cases, and glomerulosclerosis and nephrocalcinosis for 4.6% of cases (111). Nephrectomy was carried out before dialysis in 21 (32.3%) patients and after dialysis in six patients. The indications of nephrectomy were severe hemorrhage in six patients, renal cancer in eight patients, and AML in 13 patients. Eight patients died: six related to dialysis, one related to untreated uremia, and one due to septic shock. The underlying pathology of end-stage renal failure in 10 patients with TSC treated in Europe was AML alone in one patient, four had PCK, one had AML with cysts, two had atrophic kidneys with cysts, one patient had adenocarcinoma, and in one patient pathology was unidentified (112).

Other studies have provided insights into the causes of renal impairment. In a cohort study documenting deterioration of renal function in TSC patients with RAML, there was a significant difference in CKD between patients who had embolization and those who did not have (108). A confounding factor of the association of larger RAML with CKD is that larger lesions are subjected to more interventions including higher embolization rate and nephrectomy (109). Another confounding factor is the association with renal cysts either as a part of RAML pathology or associated with adult PCK mutation. In an epidemiologic study of patients with TSC, patients who consented to have an ultrasound evaluation, 69% had RAML and 30% had renal cysts (115). In a cross-sectional study of asymptomatic patients with TSC, 61% of patients had a renal lesion, 49% had AML, 32% had renal cysts, and 2.2% had renal carcinoma (90). The patients with any renal lesions had cysts alone or associated with AML in 52.9%. Out of 45 patients who had a test for serum urea and creatinine, only four had elevated levels—three of them had cysts with or without AML lesions and one had only AML. The authors suggested that the presence of renal cysts contributed to the renal dysfunction, which was also endorsed by previous reports (116, 117). A possible mechanism is the concomitant deletions affecting the TSC2 and PKD1 genes on chromosome 16p (118).

Some reports indicated that intervention was not associated with renal impairment, while others showed that inter vention/surgery rather than the AML lesions was associated with deterioration of renal function (119, 120). The discrepancy may be related to the relative proportions of sporadic versus TSC-RAML, where most of the reports associating renal impairment with RAML involve TSC patients.

Analysis of data from the above articles may lead to the conclusion that indeed renal impairment is associated with RAML, particularly in the TSC patients. The underlying mechanisms, however, might be disputed. Renal cysts alone or with AML were the most common cause that contributed to renal failure, followed by nephrectomy and intervention. A smaller number of patients may have had renal impairment because of RAML alone; however, no evidence explaining the underlying pathogenesis was provided. The significance of this observation is that treatments that target reduction of renal failure in patients with RAML should primarily aim at the reduction of nephrectomy and intervention complications. Several novel approaches including mTOR inhibition may achieve this goal. Some patients will benefit from mTOR inhibition by reducing the size of RAML. It remains to be seen if any treatment may modify the progression of renal cysts which may have a greater impact on the development of renal failure.

## Risks Related to Management

RAML may prompt intervention or surgery as a preventive or therapeutic measure. A prospective cohort study reported an intervention in 48.3% of patients with sRAML (29). The primary surgical intervention was indicated mainly for patients with fat-poor RAML (76.3%) and for lesions >4 cm; PN constituted 65.8% of the procedures. Primary embolization was scarcely used in the cohort (4.6%). Before January 2000, incidentally presenting lesions constituted 27.6% of patients and surgical extirpation constituted 28.6% of interventions; following that date, incidental presentation increased to 72.4% and surgery to 48.6%. Another study of patients with RAML reported 50.8% active treatment in the form of surgical intervention (79.8%) or embolization (20.2%) (120). At a mean follow-up of 64.8 months, 5.5% required additional intervention because of growth of lesions or hemorrhage. Others reported 48.3% interventions, 79.3% of which were PN or total nephrectomy (1). The indications for intervention were hemorrhage, pain, or suspicion of malignancy. Patients with TSC-RAML are particularly prone to such treatments. In a retrospective study of patients with TSC-RAML aged 18 years or older, 25% received an intervention. The reported rates included 17.1–18.5% embolization, 5.1–7.7% PN, 4.3–11.4% nephrectomy, 6.8–7.7% repeat embolization, and 1.7–4.5% repeat PN (121). These figures were reported before the use of mTOR inhibitors to treat similar patients.

### Nephrectomy and PN

In the early days, surgical excision in the form of nephrectomy or PN of RAML was common for the management of symptomatic cases (122), tumor hemorrhage, retroperitoneal hemorrhagic emergency (123), suspicion of malignancy (32), and later for prophylaxis. Surgical excision remains the only treatment that completely rids of the offending lesion, although recurrence from other parts of the kidney may occur. Nephrectomy or PN leads to loss of renal tissue, but in an emergency, a nephrectomy could be lifesaving. Preoperative embolization of the offending lesion may permit resuscitation and a well-planned PN and reduce the difficulty and complications of laparoscopic PN (124–126).

NSS was advocated for RAML at risk of bleeding for prophylaxis and for the suspicion of malignancy. PN, whether open surgical, laparoscopic, or robotic assisted, became the common urologic procedure. The challenges of surgery are related to high vascularity of RAML, producing difficulty that may lead to total nephrectomy and renal impairment that may develop with longer warm ischemia time. Several early series were reported utilizing NSS for RAML (33, 36, 127). Heidenreich et al. reported a summary of 10 series involving 101 patients prior to 2002 including their own (33). The indications for surgery were suspicion of malignancy, symptoms, or risk of bleeding. Early postoperative complications included a urinary leak in a minority of patients that required a secondary procedure. Renal function was maintained and no local recurrence was reported. An early series of open PN (*n* = 58, median size 3.9 cm) was reported including seven patients with multiple ipsilateral lesions, two patients with bilateral lesions, and four patients with solitary kidneys (36). Intraoperative blood transfusion was needed in 23% of cases. The early postoperative complication rate was 12.1%, most commonly due to ileus (8.6%) and urine leak (5.2%). At a median follow-up of 8 years, there were two AML recurrences (3.4%), no significant difference between preoperative and last serum creatinine was reported, and no *de novo* renal impairment was developed. A review of more contemporary series of NSS (2000–2010), open and laparoscopic, for RAML including 185 patients, reported higher complication rate (10.7–21.4% at mean follow-up of 26.4–96 months) and recurrence (3.4–5.9% at mean follow-up of 56.4–96 months) (4). Of these patients, 28 were also included in a previous review (33). Laparoscopic PN has similar surgical outcomes when performed for RAML in comparison to other renal tumors (38). At a median follow-up of 15 months, no AML recurrence or bleeding occurred. Lesion enucleation of large RAML (*n* = 31, range 9–15 cm) was carried out using an open technique (128). There was no significant change in perioperative creatinine. No complications or recurrences were reported. Retroperitoneal laparoscopy with enucleation of the lesions was described to minimize complications (129).

Robotic-assisted PN further facilitates the surgical procedure. In a multicenter study of robotic PN for patients with large RAML (*n* = 40, median diameter 7.2 cm) and challenging anatomical characteristics (median nephrometry score 9), there were minimal perioperative complications and excellent renal function preservation (41). Preoperative embolization did not affect the outcome of the procedure. In another series reporting long-term follow-up of robotic PN for RAML (*n* = 23, median size 5.2 cm), at a median of 40 months follow-up, 86.9% of GFR was preserved and no long-term complications requiring secondary intervention or AML recurrence were reported (40). Robotic PN for smaller lesions (*n* = 53, median size 2.8 cm) included lesions suspicious for malignancy (79.2%), at risk of bleeding, or causing pain (39). Similarly, the procedure was associated with excellent preservation or renal function (median 91% preservation of GFR), no surgical complications requiring secondary intervention or recurrence of lesions.

In summary, NSS is associated with no or minimal recurrence of lesions, maintenance of renal function, and few manageable complications.

### Embolization

A prophylactic embolization of lesions at high risk for bleeding became more common. The procedure is minimally invasive and has a high success rate (22, 42). Frequent retreatment, however, may be needed in nearly half of the patients and postembolization syndrome affecting as high as 86% (22). A recent review of contemporary series of embolization for RAML was reported (4). Seven articles from 2009 to 2014 including 196 patients with a large mean lesion size ranging 7.8–15 cm reported a complication rate of 0–19.5%. Repeat embolization was needed in 5.9–56.3% of cases and surgery in 0–14.8% at a mean follow-up of 14–85.2 months. In a meta-analysis of patients who received selective embolization for RAML followed for 39 months, self-limiting postembolization syndrome occurred in 36%, another morbidity in 7%, and repeat embolization or surgery was needed in 21% (130). The recurrence rate might be higher in TSC patients (131). Loss of renal function consequently may be encountered. Several reports of embolization of RAML, however, reported no deterioration of renal function (23, 24, 132). Even with preexisting renal impairment, there was no significant change in renal function after embolization alone or associated with NSS (132). In another study, in patients with TSC-RAML, there was no significant deterioration of renal function attributed to prophylactic embolization (49). The presence of contralateral normal functioning kidney may mask renal damage occurring on the embolized kidney. To answer this question, a prospective study evaluated split renal function before and after embolization using radioisotope renography and found no significant change of renal function on the treated kidney (133).

### mTOR inhibitors

Everolimus was approved for the treatment of TSC-associated RAML. No serious AE was encountered that required cessation of treatment in the clinical trials (2, 77). AE included stomatitis, acne-like skin lesions, amenorrhea, irregular menstruation, and hypercholesterolemia. Open label extension of the clinical trial showed that everolimus is well tolerated and AE decreases over time (57). Other studies confirm the efficacy and safety of everolimus with manageable AE profile (134, 135). Lower doses were also shown to be effective. The significant reduction of lesion size was maintained for 36 months on a dose of 2.5–5 mg (136). A major concern for treating physicians is how long should the treatment continue as the effect of mTOR inhibition is reversible (75, 137). To continue the treatment for RAML, consideration should be given to long-term complications and the cost compared with other treatment modalities. The treating physician needs to observe metabolic complications that may cause dyslipidemia, hyperglycemia, immunosuppression-related complications, and fertility concerns and guide therapy accordingly (138). Several studies assessed the cost of health care for patients with TSC-RAML prior to approval of everolimus and found a high cost related to renal event management (108, 139, 140). A retrospective analysis of commercially insured and Medicaid patients in the United States compared claims related to TSC-RAML to controls between 2000 and 2012 (141). TSC was associated with higher incidence of hematuria, renal hemorrhage, CKD, renal failure, and hospital mortality than patients without TSC. The most significant differences were found in patients older than 18 years and for the incidence of hematuria and CKD. The health care annual cost was significantly higher for patients with TSC-RAML versus controls (142). Currently, it is not clear how everolimus treatment may impact the cost of TSC-RAML in the long term.

## Risk of Associated Conditions

TSC-RAML, unlike its sporadic counterpart, is associated with a myriad of conditions that pose a significant risk to patients (143). Seizures, hydrocephalus, and intellectual disability are among the first presenting symptoms. The underlying neurological pathology includes cortical tubers, subependymal nodules, and giant cell astrocytomas. Other serious conditions that are commonly found are lymphangioleiomyomatosis of the lungs, cardiac rhabdomyoma, and hepatic AML. A cohort of adult women with TSC recruited by the NIH for the study of LAM had many patients with respiratory complications (75%), seizers (54%), renal intervention (43%), and mortality (12%) (144).

## Conclusion

RAML encompasses a wide spectrum of small lesions that need not be followed to massive bilateral lesions that pose life-threatening risks to the patient. The disease itself or its treatment may contribute to the development of complications. With the development of interventional treatments, minimally invasive surgery, and oral targeted therapy, there is hope that such complications can be reduced to a minimum and there will be better longevity and quality of life for patients even with the most significant disease burden.
